# Investigating the optimum size of nanoparticles for their delivery into the brain assisted by focused ultrasound-induced blood–brain barrier opening

**DOI:** 10.1038/s41598-020-75253-9

**Published:** 2020-10-26

**Authors:** Seiichi Ohta, Emi Kikuchi, Ayumu Ishijima, Takashi Azuma, Ichiro Sakuma, Taichi Ito

**Affiliations:** 1 Center for Disease Biology and Integrative Medicine, The University of Tokyo, 7-3-1 Hongo, Bunkyo-ku, Tokyo, 113-8655 Japan; 2 Department of Bioengineering, The University of Tokyo, 7-3-1 Hongo, Bunkyo-ku, Tokyo, 113-8656 Japan; 3 Department of Precision Engineering, The University of Tokyo, 7-3-1 Hongo, Bunkyo-ku, Tokyo, 113-8656 Japan; 4 Present Address: Institute of Engineering Innovation, The University of Tokyo, 7-3-1 Hongo, Bunkyo-ku, Tokyo, 113-8656 Japan

**Keywords:** Nanomedicine, Drug delivery

## Abstract

The blood–brain barrier (BBB) has hampered the efficiency of nanoparticle delivery into the brain via conventional strategies. The widening of BBB tight junctions via focused ultrasound (FUS) offers a promising approach for enhancing the delivery of nanoparticles into the brain. However, there is currently an insufficient understanding of how nanoparticles pass through the opened BBB gaps. Here we investigated the size-dependence of nanoparticle delivery into the brain assisted by FUS-induced BBB opening, using gold nanoparticles (AuNPs) of 3, 15, and 120 nm diameter. For 3- and 15-nm AuNPs, FUS exposure significantly increased permeation across an in vitro BBB model by up to 9.5 times, and the permeability was higher with smaller diameter. However, in vivo transcranial FUS exposure in mice demonstrated that smaller particles were not necessarily better for delivery into the brain. Medium-sized (15 nm) AuNPs showed the highest delivery efficiency (0.22% ID), compared with 3- and 120-nm particles. A computational model suggested that this optimum size was determined by the competition between their permeation through opened BBB gaps and their excretion from blood. Our results would greatly contribute to designing nanoparticles for their delivery into the brain for the treatment of central nervous system diseases.

## Introduction

Nanoparticles have attracted global attention in the biomedical field. It has been revealed that their interaction with cells and/or tissues can be tailored through nanoparticle design, such as their size, shape, and surface chemistry^1^. Combined with the advances in nanoparticle functionalization methods, this has opened the way for various biomedical applications of nanoparticles, including drug delivery, imaging, and therapies^2,3^. However, despite the promise of nanoparticle-based systems, their translation to clinical use remains a challenge, mainly due to the low efficiency of their delivery to target sites^4,5^. Various factors have been proposed as hampering nanoparticle delivery, including uptake by the reticuloendothelial system (RES), restricted diffusion in dense extracellular matrix (ECM), resistance by interstitial pressure, and clearance via the renal system^6–8^.

The brain is one of the most difficult target organs to deliver nanoparticles to because of the existence of the blood–brain barrier (BBB). The BBB is composed of brain endothelial cells attached to a continuous basement membrane and linked together by tight junctions that prevents foreign substances from entering into the brain^9^. Even small molecular drugs can barely cross the BBB, which is a major limitation for the treatment of central nervous system (CNS) diseases, such as Alzheimer’s disease and Parkinson’s disease; diseases whose prevalence is rapidly increasing as societies around the world are aging. In the case of nanoparticles, the restriction of their permeation across the BBB is even more pronounced because of their relatively large size. Although various delivery methods have been attempted, e.g., using receptor-mediated endocytosis^10–12^, transcytosis^13,14^, or transporters^15,16^, the efficiency of delivering nanoparticles into the brain is insufficient to fully exploit their therapeutic and diagnostic potential. For example, using transferrin receptor-targeted nanoparticles is one of the most widely used strategies to get nanoparticles across the BBB, but it typically results in < 0.1% delivery efficiency to the brain^10^.

Focused ultrasound (FUS) in combination with the administration of microbubbles (MBs) is an emerging technique being investigated to enhance the permeation of therapeutics across the BBB in a noninvasive, localized, and transient manner^17^. FUS induces inertial or stable cavitation with MBs that exerts a mechanical force onto capillary walls, leading to a temporary opening of the BBB via the widening of tight junctions^17–19^. The enhanced delivery of small molecular drugs^20,21^, oligonucleotides^22,23^, and antibodies^24–26^ into the brain via FUS-induced BBB opening has been demonstrated in vivo. In addition, clinical trials are now being conducted into the FUS-assisted delivery of small molecular drugs into gliomas^27,28^. This technology could provide a promising strategy for improving the efficiency of nanoparticle delivery to the brain, although there are still only a limited number of reports on its application for nanoparticles^29–34^.

**Figure 1:**
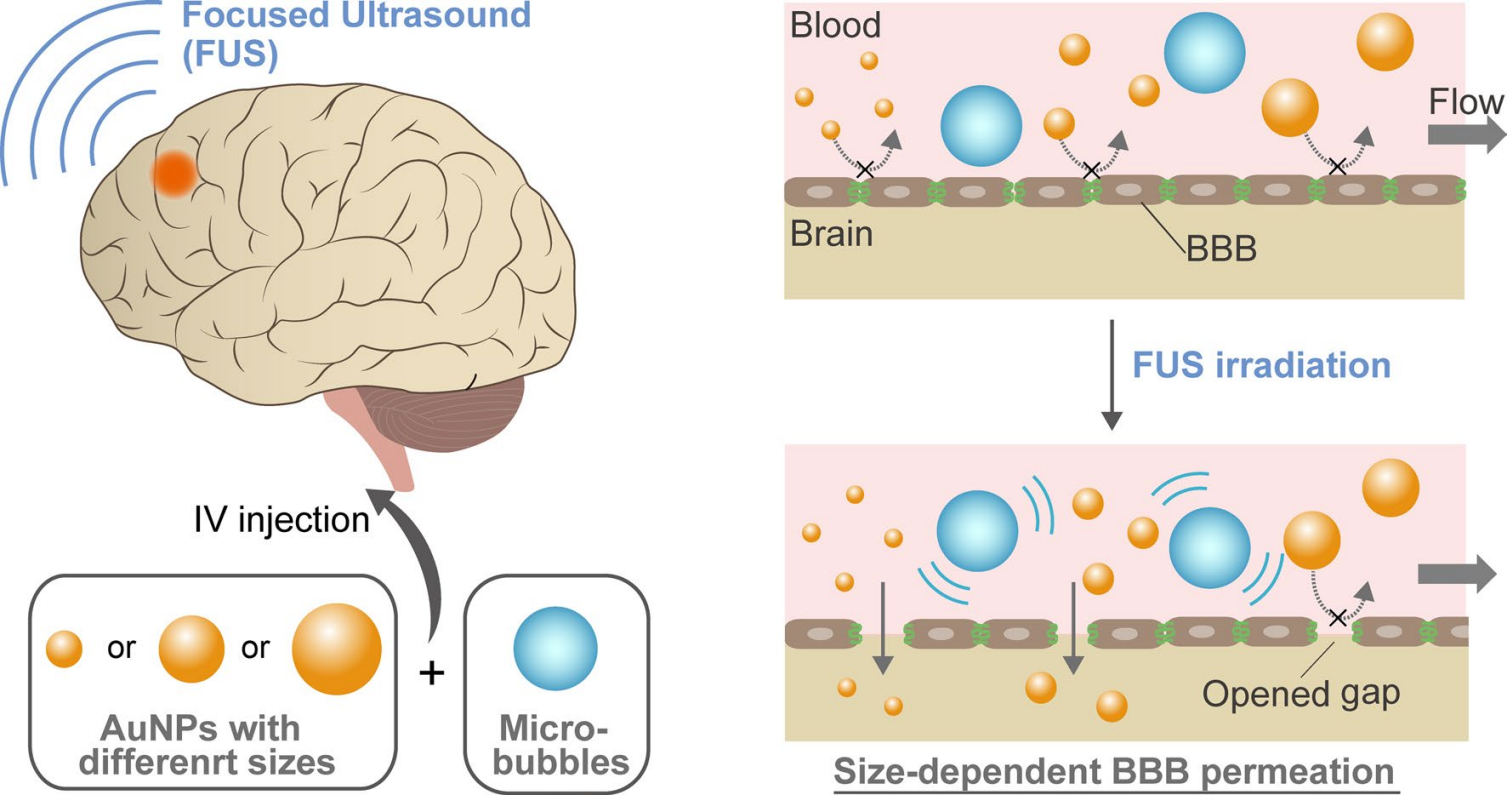
Size-dependent delivery of nanoparticles into the brain assisted by FUS-induced BBB opening. Under the normal state, nanoparticles entrance into the brain is strictly restricted by the BBB. By FUS exposure, inertial or stable cavitation with MBs is induced to exert a mechanical force on the BBB, which temporarily widens the tight junctions. AuNPs can cross the BBB through these opened gaps, depending on their size.

To employ FUS-induced BBB opening for nanoparticle delivery, a question that must be addressed is how the size of nanoparticles can affect the enhanced permeation through opened BBB gaps. It is expected that the optimum nanoparticle design for this delivery mechanism would be different from that usually employed for enhanced permeation and retention (EPR)-based delivery strategies for tumors, in which nanoparticles are extravasated from naturally leaky blood vessels^1^. However, although some previous studies investigated the effect of the size of nanoparticles, such as liposomes, on this strategy^34^, the mechanism still needs to be clarified, especially in single to sub-hundred nanometer range. Here, we explored the effect of nanoparticle size on their delivery into the brain assisted by FUS-induced BBB opening, using polyethylene glycol (PEG)-coated gold nanoparticles (AuNPs) of different sizes, 3 to 120 nm, as a model (Fig. 1). An in vitro BBB model capable of FUS exposure was developed to examine the size-dependent permeation behavior of these particles. The size-dependent delivery of AuNPs into the brain was further investigated in vivo via transcranial FUS exposure in mice. Based on the obtained results, a kinetic model was proposed to estimate the optimum nanoparticle size for delivery into the brain assisted by FUS-induced BBB opening.

## Results and discussion

**Figure 2:**
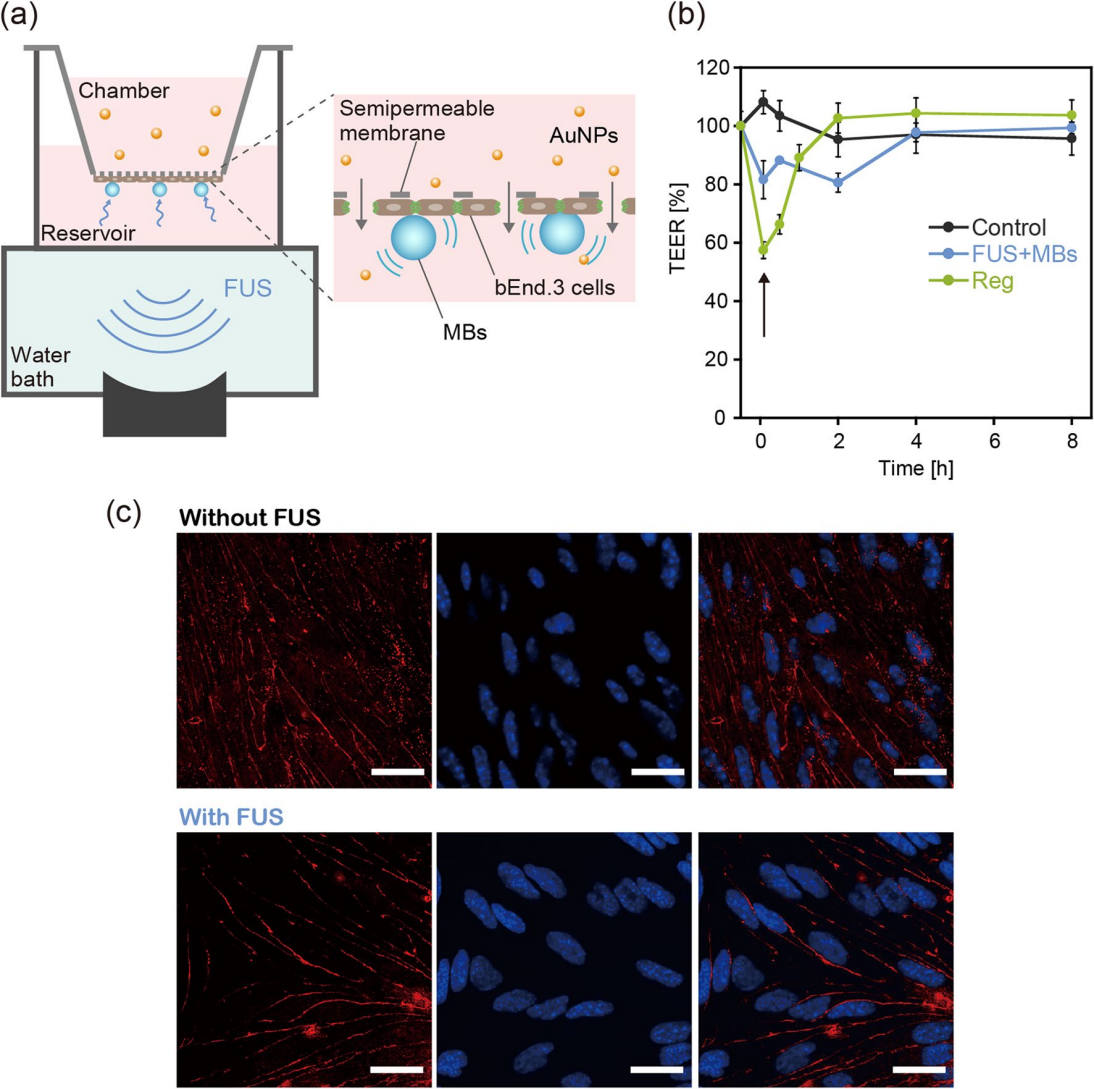
In vitro investigation of BBB opening via the integration of a semipermeable culture insert and FUS exposure setup. (**a**) A schematic diagram of the apparatus constructed for evaluating the FUS-induced enhancement of BBB permeability to nanoparticles. (**b**) Time change in TEER induced by FUS exposure in the presence of MBs. The effect of treatment with 10 μM regadenoson (Reg) was also evaluated for comparison. The arrow indicates the time when FUS exposure or Reg treatment was conducted (defined as 0 h). The control group was the BBB model that received no treatment. N = 4. (**c**) Immunohistochemical staining of the tight junction-associated protein ZO-1 (red) in bEnd.3 cells, with and without FUS exposure, observed using a confocal laser scanning microscope. Cell nuclei were also stained with DAPI (blue). The scale bar represents 10 μm.

First, we constructed an in vitro BBB model capable of FUS exposure under the existence of MBs (Figs. 2a and S1). bEND.3 mouse brain endothelial cells were chosen as model BBB cells in this study. Although the use of primary brain endothelial cells and/or co-culture with other cells would provide a physiologically more relevant environment, bEND.3 cells consistently show a stable barrier function and thus have been widely used in previous studies on BBB permeability to therapeutics^35^. The cells were cultured on a semipermeable membrane of the Transwell culture insert. The culture insert was set on a handmade culture well, which acted as a reservoir for permeates. Sonazoid microbubbles (MBs), used clinically for ultrasound diagnosis, were added to the culture well and then allowed to attach to the bEND.3 cell surface by buoyancy. The culture well was directly connected to a water bath, through which FUS was exposed for 40 s, with a burst-length of 10 ms and a repetition frequency of 1 Hz. The resonant frequency and peak-positive pressure (PPP) were 1 MHz and 88 kPa, respectively. The ultrasound beam profile at the bottom of the well is shown in Fig. S2.

To confirm if our in vitro model could replicate FUS-induced BBB opening, the time change in transendothelial electrical resistance (TEER), which indicates the integrity of tight junctions in the BBB model, was evaluated after exposure to FUS (Fig. 2b). Regadenoson (Reg), which is an A_2A_ adenosine receptor agonist that reportedly enhances BBB permeation by therapeutics^36,37^, was also used for comparison. While TEER did not change in the control group (without FUS), following FUS exposure it decreased to ca. 80% and then gradually recovered to 100% after 4 h. These results confirmed that FUS exposure temporarily decreased the BBB integrity in our in vitro model, before it gradually recovered to its original state. The addition of Reg also induced a temporary decrease in TEER, which was consistent with previous reports^37^. We noted that a reduction in TEER could be induced even without the presence of MBs (Fig. S3), although a higher acoustic pressure of FUS (PPP = 320 kPa) was required, which poses a greater risk of cellular damage. As discussed in previous reports, the oscillation of MBs can transmit mechanical forces to the BBB layer, which contribute to a decrease in the FUS intensity-threshold necessary for inducing the BBB to open^17–19^.

While partial disintegration is necessary for enhanced BBB permeability, the creation of large defects could induce possible adverse effects. Immunohistochemical staining of a tight junction-associated protein, ZO-1, was conducted to examine any structural changes in tight junctions following exposure to FUS (Fig. 2c). Compared with the control group without FUS, no obvious difference in ZO-1 expression following FUS exposure was observed within the resolution range of a confocal laser microscope (i.e., the sub-micron range). These results suggested that the small gaps at less than the sub-micron scale were created by FUS exposure in the BBB model, but not the large, micron-scale defects.

**Figure 3:**
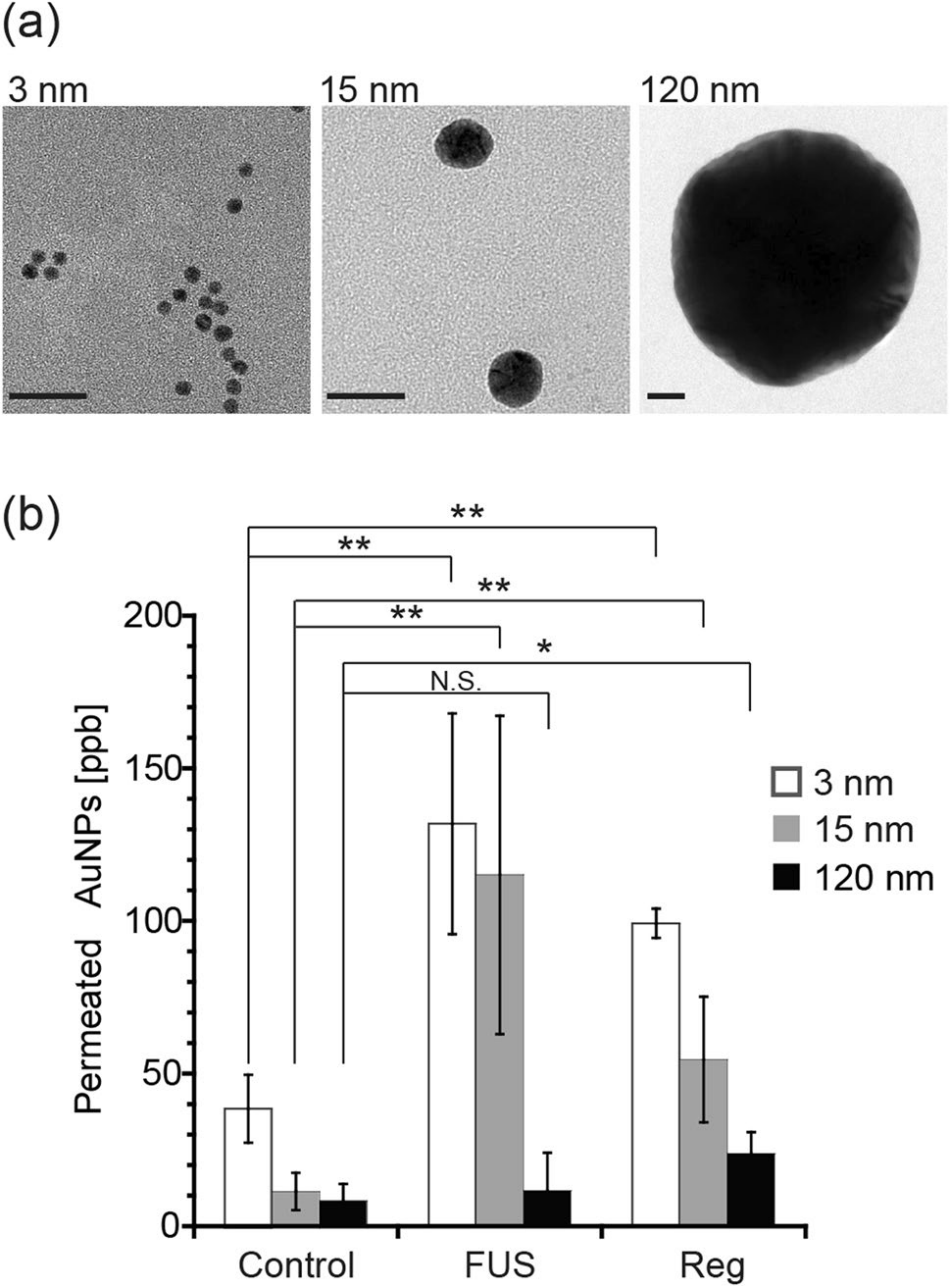
The effect of nanoparticle size on their permeability across the in vitro BBB model following its exposure to FUS. (**a**) TEM images of synthesized AuNPs with diameters of 3, 15, and 120 nm. Scale bar is 20 nm. (**b**) The amount of 3, 15, and 120 nm AuNPs that permeated across the in vitro BBB model following its exposure to FUS in the presence of MBs. The effect of treatment with 10 μM Reg was also evaluated for comparison. The control group was the BBB model without any treatment. **p < 0.01; *p < 0.05; N.S., not significant. N = 4.

AuNPs were chosen for this study because their size can be easily and precisely controlled. They are also biologically inert and nontoxic, and are therefore widely used in the biomedical field. AuNPs with diameters of 3, 15, and 120 nm were synthesized according to previously published methods^38,39^. Transmission electron microscopy (TEM) images of the synthesized AuNPs are shown in Fig. 3a. The average diameters as determined from the TEM images were 3.7 ± 0.5, 14.4 ± 1.7, and 120 ± 7.2 nm. Hydrodynamic size distributions as measured by dynamic light scattering (DLS) are also shown in Fig. S4. The obtained AuNPs were PEGylated using thiol-terminated PEGs (MW = 5 kDa) according to previously published methods^40,41^ to improve their colloidal stability and blood half-life. The summary of characterization is shown in Table S1. While zeta potential of AuNPs before PEGylation was − 28 to − 38 mV due to negatively charged citrate on the surface, it was increased to − 6 to − 9 mV after PEGylation. The surface densities of conjugated PEG were 1.1, 1.6, and 1.3/nm^2^ for 3, 15, and 120 nm AuNPs, respectively. These results were consistent with previous works^40,41^, suggesting the successful PEGylation of AuNPs with all sizes. The cytotoxicity of the obtained AuNPs was further tested via cell viability assay using HUVECs (Fig. S5). No significant decrease in cell viability was observed within the examined concentration range for AuNPs with all sizes, suggesting their good biocompatibility.

The AuNPs obtained were applied to the in vitro BBB model to investigate the effect of nanoparticle size on BBB permeation assisted by FUS exposure (Fig. 3b). The amount of AuNPs permeating from the chamber to the reservoir through the model BBB layer was quantified via ICP-OES. The amount of AuNPs that permeated across the BBB was significantly increased, by 3.4 and 9.5 times for 3 and 15 nm AuNPs, respectively, following FUS exposure. However, FUS exposure induced no significant difference in the permeation of 120 nm particles. The amount of AuNPs that permeated the BBB increased with decreasing AuNP size. Similar trends were also observed following FUS exposure at higher acoustic pressures without MBs (Fig. S6). These results suggested that the sizes of the gaps that opened in the BBB model were between approximately 15 and 120 nm. These results are consistent with the previous in vivo experiments using dextran with different molecular weights (3 to 2000 kDa), in which the opened BBB gaps ranged from 2.3 to 54.4 nm depending on the acoustic pressure^42,43^. It was considered that since smaller permeates have a lower permeation resistance in narrow channels when the sizes of permeates and channels are comparable^44,45^, smaller AuNPs showed a higher tendency for permeability through gaps opened in the BBB.

**Figure 4:**
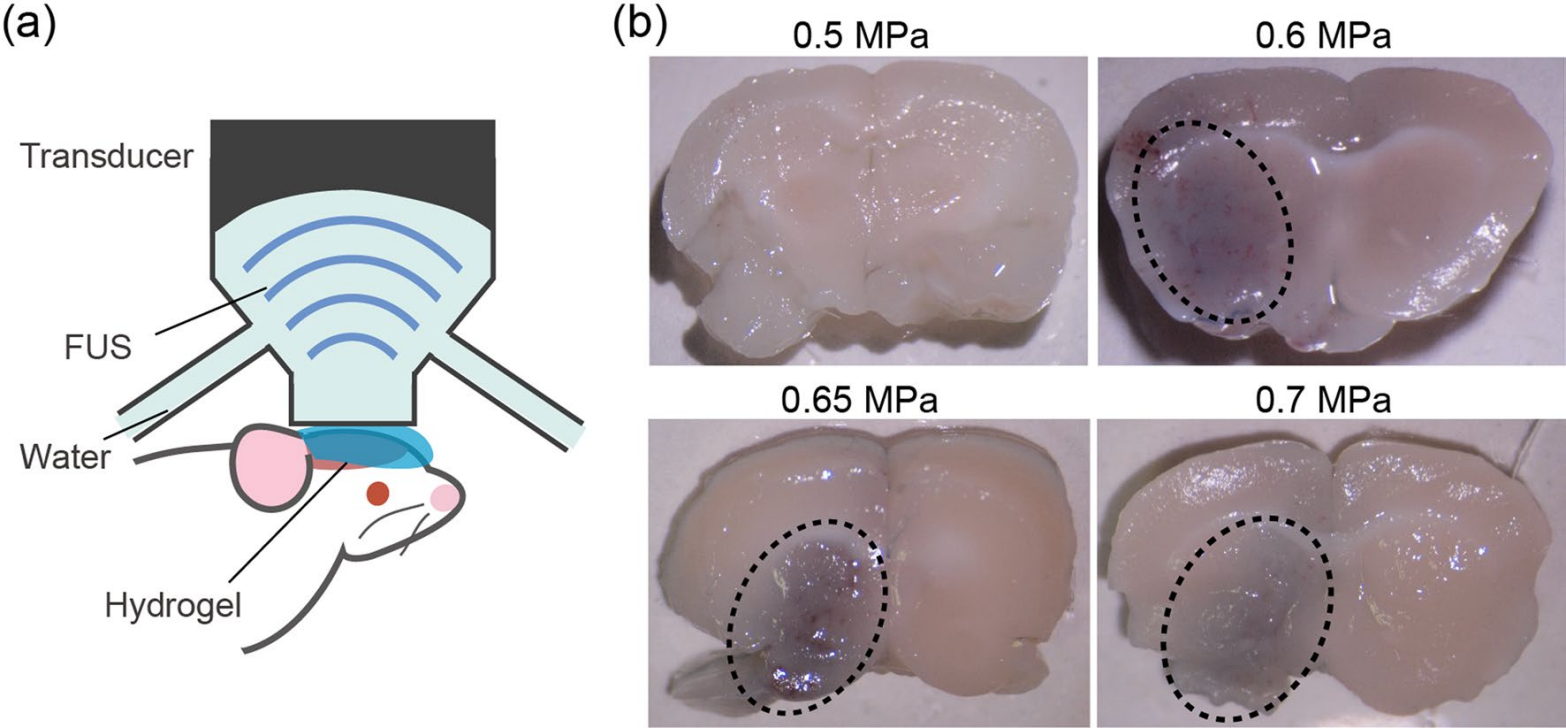
In vivo investigation into opening the BBB via intracranial FUS exposure. (**a**) A schematic diagram of the experimental setup. (**b**) Brain sections of mice subjected to FUS of different acoustic pressures. Only the left side of the brain was exposed to FUS. Dotted circles indicate areas where the permeation of trypan blue was observed. The dose of MBs was 8.0 × 10^7^ bubbles/mouse.

FUS-induced BBB opening was further examined in vivo using a transcranial FUS exposure setup (Figs. 4a and S7). A mouse was set on a mechanical stage equipped with an FUS transducer which had an adjustable focal position. FUS was exposed intracranially right after the administration of MBs and materials. The ultrasound beam profile for this setup is shown in Fig. S8. To determine the appropriate FUS conditions to open the BBB, the mouse brain was first exposed to FUS beams of different acoustic pressures. Together with MBs, trypan blue was also injected to confirm the BBB opening. Only one side of the brain was subjected to FUS, so that the exposed area could be distinguished. Figure 4b shows sections of mouse brain following the treatment. No permeation of trypan blue was observed at acoustic pressures of less than 0.5 MPa. However, at pressures exceeding 0.6 MPa, a blue-stained area was observed around the FUS focal point, indicating that the BBB had been successfully opened. This acoustic pressure threshold could be adjusted by altering the dose of MBs: a higher dose of MBs reduced the threshold pressure necessary for opening the BBB (Fig. S9). It should be mentioned that if the acoustic pressure exceeded 0.8 MPa, FUS exposure caused severe bleeding in the brain (Fig. S10). Therefore, it is essential to choose an appropriate FUS condition to achieve opening of the BBB while still being noninvasive. Based on these results, an acoustic pressure of 0.5–0.7 MPa and an MB concentration of 8.0 × 10^7^–8.0 × 10^8^ bubbles/mouse were used for the following experiments.

**Figure 5:**
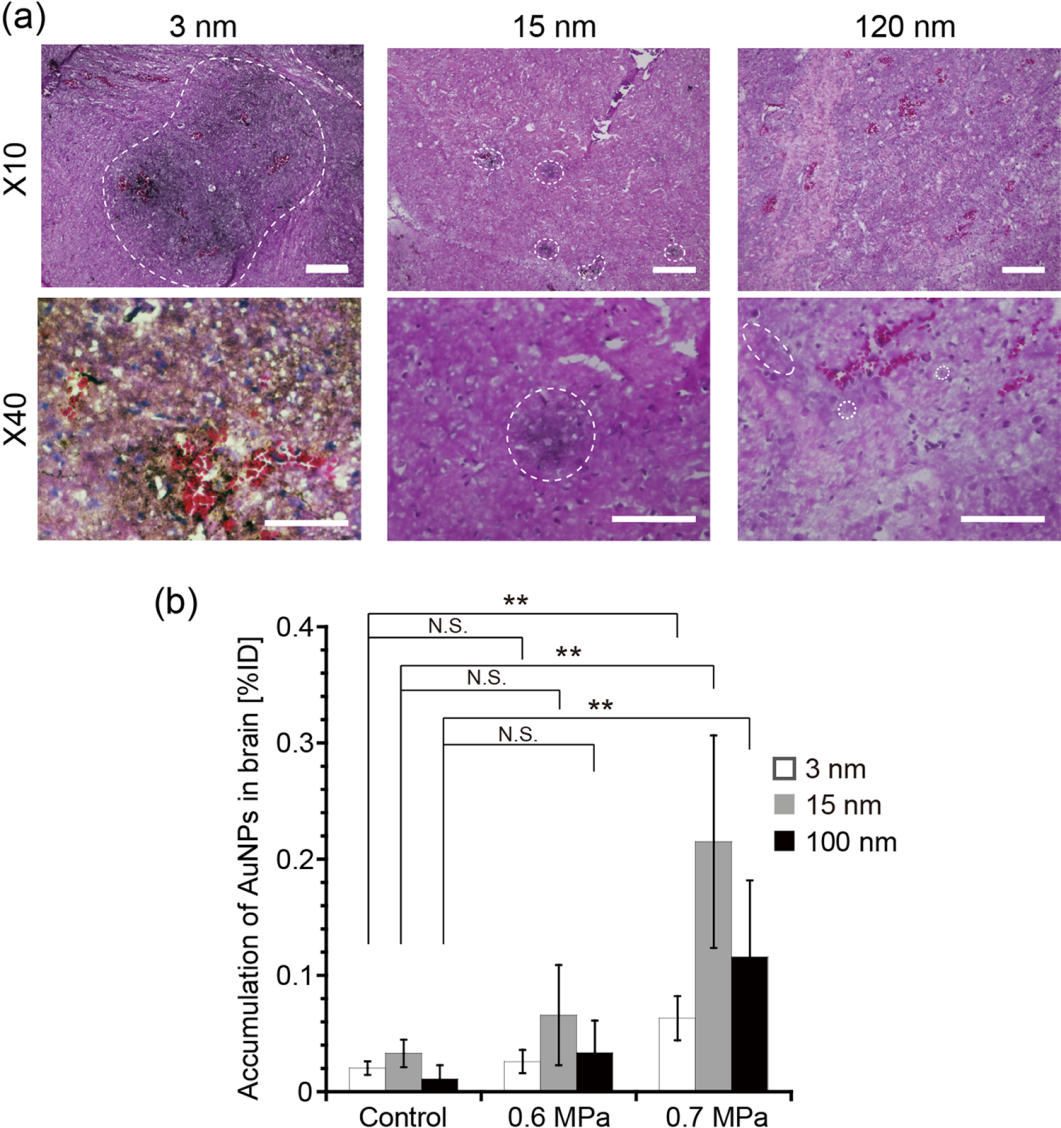
The effect of nanoparticle size on their delivery into the brain assisted by FUS-induced BBB opening in vivo. (**a**) Distribution of 3, 15, and 120 nm AuNPs in mouse brains after treatment with FUS. Brain sections were stained using HE and silver staining. The scale bar represents 200 μm for × 10 images and 100 μm for × 40 images. Dotted circles indicate areas where delivered AuNPs were detected. Note that 3 nm AuNPs were found throughout the × 40 image and thus no dotted circle is depicted. (**b**) The amount of 3, 15, and 120 nm AuNPs delivered into mouse brains when assisted by FUS-induced BBB opening. Acoustic pressures of 0.6 and 0.7 MPa were used. The dose of MBs was 8.0 × 10^7^ bubbles/mouse. **p < 0.01; *p < 0.05; N.S., not significant. N = 4.

Using the above optimized conditions, the delivery into the brain of AuNPs of different sizes was examined via FUS-induced BBB opening. Figure 5a shows hematoxylin–eosin (HE) and silver stained images of mouse brains. AuNPs of all sizes were observed in the brains, demonstrating their successful delivery via FUS-induced BBB opening. It was also found that smaller AuNPs were more widely distributed throughout the brains, presumably due to their lower diffusion resistance in brain tissue following their extravasation from blood vessels. The wider distribution of smaller nanoparticles in tissues was also reported for the delivery to tumors via the EPR effect, although the diffusion distance was shorter in that case because of the dense ECM network^46,47^. The amount of AuNPs delivered was further quantified using ICP-OES (Fig. 5b). The delivery efficiency increased with increasing acoustic pressure (Figs. 5b and S11). Following exposure to an FUS of 0.7 MPa, the amount of AuNPs delivered significantly increased for all sizes: 3, 15, and 120 nm AuNPs showed increased brain accumulation of 3.1, 18.2, and 5.4 times, respectively, compared with the control group. When comparing the different sizes, the medium-sized, 15 nm AuNPs showed the highest delivery efficiency (0.22% ID), which differed from that observed in the in vitro BBB permeation experiment (Fig. 3). A similar size dependency was also observed for higher doses of MBs (Fig. S12). These results suggested that smaller particles are not necessarily better, and that there is an optimum size of nanoparticles to use for their delivery into the brain assisted by FUS-induced BBB opening.

We hypothesized that the optimum size of nanoparticles for FUS-assisted delivery into the brain is determined by competition between permeation across the gaps opened in the BBB and excretion from blood circulation. While smaller particles are better for permeating through gaps opened in the BBB as demonstrated by the in vitro BBB model (Fig. 3), small particles in the single-nm range are rapidly excreted from blood circulation via renal clearance^8,48^. Therefore, medium-sized particles (i.e., 15 nm in our experiments) that can address both of these issues would be optimal for this delivery strategy.

**Figure 6:**
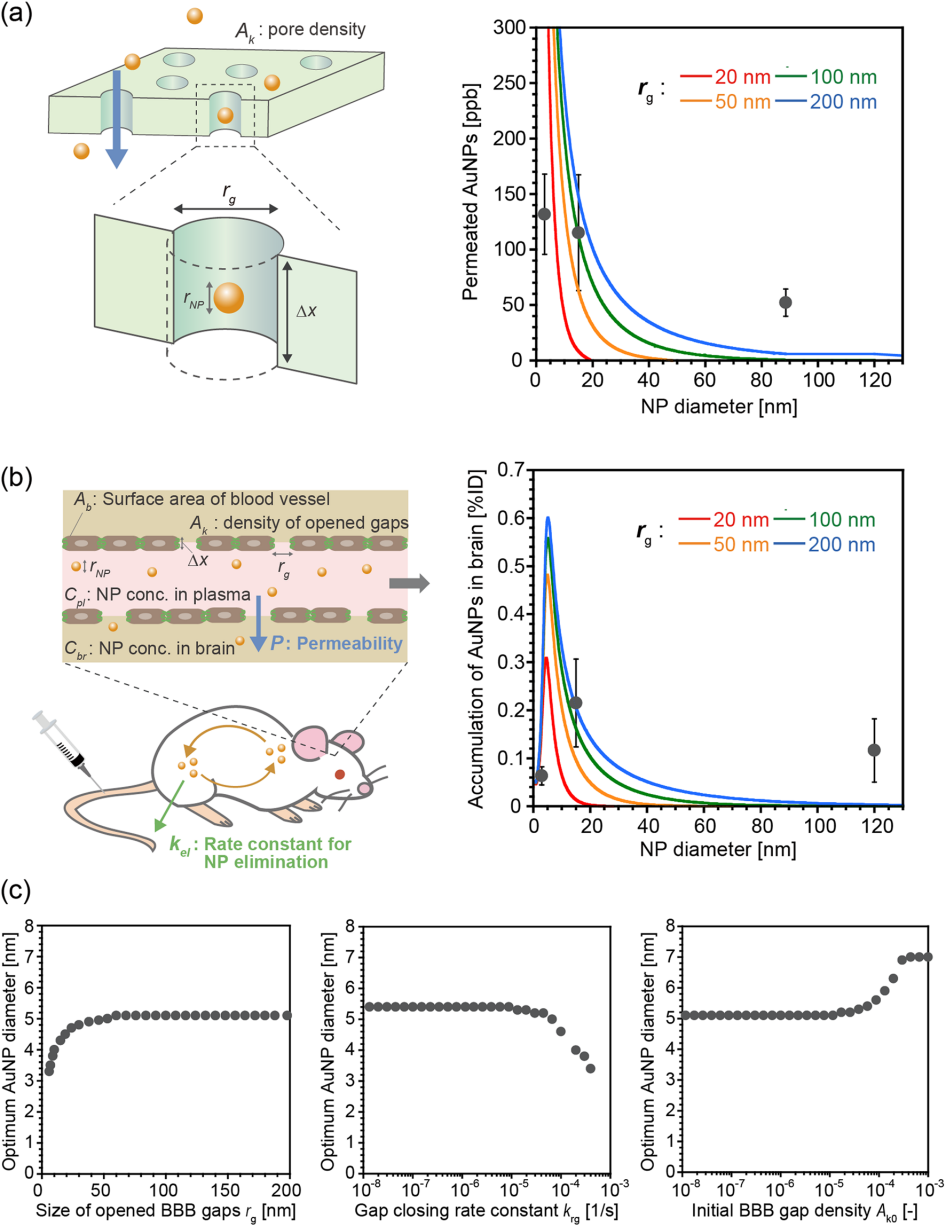
Kinetic modeling of FUS-assisted nanoparticle delivery into the brain. (**a**) Calculation of size-dependent BBB permeation of AuNPs based on a modified pore-flow model. The sizes of gaps opened in the BBB were assumed to be 20, 50, 100, and 200 nm. Other parameters used for the calculation are summarized in Table S1. (**b**) Calculation of size-dependent delivery of nanoparticles into the brain assisted by FUS, via the coupling of the BBB permeability model with nanoparticle elimination kinetics. The sizes of the gaps opened in the BBB gaps were assumed to be 20, 50, 100, and 200 nm. Other parameters used for the calculation can be found in Table S1. (**c**) Effect of *r*_g_, *k*_rg_, and *A*_k0_ on the optimum AuNP size for FUS-assisted nanoparticle delivery into the brain. The diameter of AuNPs that showed the highest delivery efficiency at each calculated condition was plotted against *r*_g_, *k*_rg_, and *A*_k0_. *r*_g_ = 100 nm was used for the calculations to examine the effect of *k*_rg_ and *A*_k0_.

Based on the above hypothesis, we modeled the kinetics of FUS-assisted nanoparticle delivery into the brain. Assuming that the gaps opened in the BBB are straight pores of uniform size *r*_g_, the size-dependent BBB permeation of nanoparticles can be modeled using the pore-flow model^44,45^, with modification, which has previously been used to model the filtration across biological and artificial membranes (Fig. 6a). The nanoparticle concentrations inside and outside of the chamber, *C*_in_ and *C*_out_, can be described by the following equations using permeability, *P*^44,45^:

1 $$ V_{out} \frac{{dC_{out} }}{dt} = A_{m} P\left( {C_{in} - C_{out} } \right) $$

2 $$ V_{in} C_{in0} = V_{in} C_{in} + V_{out} C_{out} $$

where *V*_in_ and *V*_out_ represent the volumes of culture media inside and outside of the chamber, *A*_m_ is the area of the semipermeable membrane, and *C*_in0_ is the initial nanoparticle concentration in the chamber. In this model, *P* can be described as a function of the size ratio of particles to pores, *q*, as follows^44,45^:

3 $$ P = D \cdot f(q) \cdot S_{D} (q)\left( {\frac{{A_{k} }}{\Delta x}} \right) $$

4 $$ f(q) = \frac{{1 - 2.100q + 2.086q^{3} - 1.7068q^{5} + 0.726q^{6} }}{{1 - 0.758q^{5} }} $$

5 $$ S_{D} (q) = \left( {1 - q} \right)^{2} $$

6 $$ q = \frac{{r_{NP} }}{{r_{g} }} $$

where *r*_NP_ and *△x* represent the radius of the nanoparticles and the thickness of the pores, respectively, and *D* represents the diffusion coefficient of the nanoparticles, which was calculated as a function of *r*_NP_ using the Stokes–Einstein law, as follows:

7 $$ D = \frac{kT}{{6\pi \eta (2r_{NP} )}} $$

where *k*, *T*, and *η* are the Boltzmann constant, temperature, and viscosity of the media, respectively. The decrease in *r*_g_ and pore density *A*_k_ with time, which corresponds to the recovery of opened BBB gaps, was modeled using a first-order kinetic law as follows:

8 $$ r_{g} = r_{g0} \exp \left( { - k_{rg} t} \right) $$

9 $$ A_{k} = A_{k0} \exp \left( { - 2k_{rg} t} \right) $$

where *k*_rg_ is the rate constant for the closing of the gaps. (The details are described in the Supporting Information). Figure 6a shows the calculated size-dependence of permeability *P* with different sizes of BBB gaps, *r*_g_. Permeability decreased with larger nanoparticle sizes and smaller BBB gaps. The calculated size dependencies fitted approximately with the in vitro experimental results shown in Fig. 3b. However, because of the simplicity of the model, it was difficult to obtain a perfect fit using a single curve. Modeling of more detailed factors, such as the size distribution of gaps opened in the BBB, would improve the accuracy of the calculation. The effects of other parameters are shown in Figure S13.

To describe the in vivo delivery kinetics, the BBB permeation model described above was coupled with size-dependent elimination of nanoparticles from the bloodstream (Fig. 6b). We compiled previously reported blood half-lives of PEGylated nanoparticles, regardless of the type of materials used^8,47,49–51^, and then plotted against their size (Fig. S14). It was found that the size dependency of the blood half-life could be fitted with two master curves, which respectively corresponded to renal clearance and RES uptake. In the case of single-nm particles, since these particles are rapidly excreted via the kidneys, their blood half-life is dominated by their renal clearance rate, which decreases with decreasing particle size^8,48^. Conversely, when the particle size exceeds a particular threshold, RES uptake occurs more rapidly than renal clearance. In this latter case, because larger particles are taken up more easily by the RES, the blood half-life decreases with increasing particle size^47^. By combining this size dependency of the blood half-life with Eqs. 3–9, the kinetics of nanoparticle accumulation in the brain can be described as follows:

10 $$ V_{b} \frac{{dC_{b} }}{dt} = P \cdot A_{b} C_{pl} $$

11 $$ V_{pl} \frac{{dC_{pl} }}{dt} = - P \cdot A_{b} C_{p} - k_{el} (r_{NP} ) $$

where *k*_el_(*r*_NP_) is the rate constant for AuNP elimination as a function of *r*_NP_; *V*_b_ and *V*_pl_ represent the volume of the brain and plasma, respectively; *C*_b_ and *C*_pl_ represent the AuNP concentration in the brain and plasma, respectively; and *A*_b_ represents the area of blood vessels in the brain. Figure 6b illustrates the calculated size dependency of nanoparticle accumulation in the brain, assuming different sizes of gaps opened in the BBB. The calculated delivery efficiencies showed similar trends to the in vivo experimental results in Fig. 5b and exhibited a maximum value at a particular nanoparticle size. The optimum nanoparticle size increased with increasing size of gaps opened in the BBB, from 5.0 nm for 50 nm gaps to 6.2 nm for 200 nm gaps (Fig. 6c). The optimum size also increased with decreasing *k*_rg_ and increasing *A*_k0_, which corresponded to slower BBB gap closure and a higher number of open BBB gaps, respectively. The effect of other parameters is also shown in Fig. S15. Although a better fit could be obtained by considering more detailed factors, the consistency between our simple model calculations and the experimental results confirmed our hypothesis that the optimum nanoparticle size is determined by the competition between permeation through gaps opened in the BBB and excretion from blood circulation.

There have been intensive discussions on the optimum design of nanoparticles for tumor targeting via an EPR effect-based strategy. For example, 50 nm silica nanoparticles showed the highest tumor accumulation out of particles sized 20, 50, and 200 nm in a muse primary and metastatic breast cancer model^52^, whereas 30 nm micelles were reported to be optimal for a poorly permeable pancreatic tumor model in mice^46^. Although these investigations have made a great contribution to the advancement of tumor-targeted drug delivery/imaging, their findings are not applicable to target sites where leaky blood vessels are absent. Shen et al. reported that the delivery efficiency of 55 nm liposomes via the FUS-induced BBB opening strategy was higher than that of 120 nm and 200 nm^34^. In this study, we explored further smaller size range using size-controlled AuNPs. Our experimental and calculated results suggested that the optimum size of nanoparticles for delivery into the brain via this mechanism would be 5 to 6 nm in our system. This optimum size is relatively small compared with particles used for tumor delivery described in previous reports, because of the smaller gaps opened in the BBB compared with tumor blood vessels. In addition, since the observed optimum nanoparticle size is comparable with the size of certain biomolecular drugs, including anti-amyloid *β* antibodies used to treat Alzheimer’s disease^53^, our results also suggest that FUS-induced BBB opening would be useful for these agents.

We note that the above nanoparticle optimum size was obtained in healthy mice brain using AuNPs, and thus the optimum size would be changed depending on the type of disease, personal characteristics, physicochemical property of nanoparticles, as well as the conditions for FUS exposure. For example, BBB integrity is lower in the tumor site than healthy brain^54^, which would lead to higher penetration of nanoparticles. In addition, shape, rigidity and surface chemistry of nanoparticles would also affect their permeability across BBB. The effect of these variations needs to be clarified in future studies to achieve personal or disease-specific optimization of nanoparticle design for future clinical translation of this technology. For this purpose, we believe that a basic understanding of these phenomena via our simple modeling approach would be of great help in the rational design of particles.

Although the number of patients with CNS diseases, particularly neurodegenerative disorders, is increasing globally, the treatment of these diseases remains a great challenge, and, unfortunately, there is currently no effective treatment for most of them. New approaches have recently been attempted for the efficient delivery of drugs into the brain to treat CNS diseases^23,37,55,56^. For example, it has been reported that a rapid glycemic increase via fasting could dramatically enhance the delivery of glucose-modified micelles to the brain^55^. Among them, drug-carrier delivery into the brain assisted by FUS-induced BBB opening, could be a promising approach to address this unmet need because of its site-specificity, noninvasiveness, and applicability to a wide range of therapeutics. However, this technology is still currently under development, and thus further careful and detailed examinations of its safety and efficacy are required. As shown in Fig. S9, strong FUS stimulation could induce damage to the brain. While no adverse event was observed in a clinical trial of FUS-induced BBB opening for patients with recurrent glioblastoma^27^, its possibility to induce sterile inflammation in brain was also pointed out by another study using rats^57^. For brain tumor, metastasis caused by FUS-induced damage and bleeding of tumor could be a further concern. We believe that the interactive co-development of drug carriers, MBs^58–60^, and FUS equipment to create an entire system will lead to safe, novel therapeutic strategies for CNS diseases.

## Conclusion

The size-dependence of nanoparticle delivery into the brain assisted by FUS-induced BBB opening was investigated using AuNPs of 3 to 120 nm diameter. Our in vivo experiment suggested that smaller particles were not necessarily better for this delivery mechanism: medium-sized 15 nm nanoparticles showed the highest delivery efficiency into the brain (2.2% ID via 0.7 MPa FUS), compared with 3- and 120-nm nanoparticles. This size-dependence was attributed to competition between permeation through gaps opened in the BBB and excretion of particles from blood circulation. While smaller particles are preferable for BBB permeation through opened BBB gaps as illustrated by our in vitro experiment, small particles in the single-nm range are quickly removed from the bloodstream via the kidneys. Based on the above hypothesis, a computational model was proposed to estimate the optimum size of nanoparticles for FUS-assisted delivery into the brain. Our results will provide useful information for overcoming the BBB to achieve successful nanoparticle delivery into the brain for the treatment of CNS diseases.

## Materials and methods

### Materials

Gold (III) chloride (HAuCl4), sodium citrate tribasic, tannic acid, potassium carbonate, Bis(p-sulfonatophenyl)phenylphosphine dihydrate dipotassium salt (BSPP), hydroquinone, and Dulbecco’s modified Eagle’s medium (DMEM) were purchased from Sigma-Aldrich (St. Louis, MO, USA). Tween 20 was purchased from Tokyo Chemical Industry (Tokyo, Japan). Thiol-terminated metoxypolyethylene glycol (PEG-SH) (Sunbright ME-050SH, MW = 5 kDa) was purchased from NOF Corp. (Tokyo, Japan). Sonazoid was purchased from Daiichi Sankyo Co. Ltd. (Tokyo, Japan). bEND.3 mouse brain endothelial cell was provided by the RIKEN cell bank. Penicillin–streptomycin-amphotericin B and 20% formalin were purchased from Wako Pure Chemical Industries (Osaka, Japan). Fetal bovine serum (FBS) and ZO-1 rabbit polyclonal antibodies were purchased from Thermo Fisher Scientific Inc. (Waltham, MA, USA). TRITC-conjugated anti-rabbit IgG was purchased from Agilent Technologies Inc. (Santa Clara, CA, USA). DAPI was purchased from Dojindo (Kumamoto, Japan). A silver enhancement kit was purchased from BBI Solutions (Crumlin, UK).

### Synthesis and surface modification of AuNPs

Gold nanoparticles were synthesized according to previously described methods^38,39^. Briefly, 3 nm AuNPs were synthesized by first adding 1.0 ml of 1.0 wt% HAuCl_4_ to 98 ml pure water and brought to 60 °C on a stirring hotplate. A reducing solution was prepared by adding sodium citrate tribasic (1.0 wt%, 6.0 ml), tannic acid (1.0 wt%, 7.5 ml), and potassium carbonate (0.35 wt%, 7.5 ml) to 9 ml pure water. Under vigorous stirring, 20 ml of the reducing solution was injected swiftly into the HAuCl_4_ solution. The reaction was maintained at 60 °C for 30 min and then at 90 °C for 10 min. After cooling on ice, 1.0 ml of 80 mg/ml BSPP was added to the nanoparticle solution and stirred overnight to improve particle stability. The obtained AuNPs were washed three times by centrifugation using a 0.01% aqueous solution of sodium citrate containing 0.01% Tween 20.

To synthesize 13 nm AuNPs, 1.0 ml of 1.0 wt% HAuCl_4_ was added to 98 ml pure water. The solution was brought to the boil on a stirring hotplate. Then, 1.0 ml of 3.0 wt% sodium citrate tribasic was injected swiftly into the boiling solution under vigorous stirring. The reaction was allowed to proceed for 10 min, followed by cooling on ice.

120 nm AuNPs were synthesized by seed-mediated growth. 340 μl of 13 nm AuNPs (2.4 nM) were mixed with 995 μl HAuCl_4_ and 97.2 ml pure water, followed by the sequential addition of 995 μl sodium citrate tribasic (15 mM) and 995 μl hydroquinone (25 mM) under rapid stirring. The AuNPs obtained were washed three times by centrifugation using an aqueous solution of 0.01% sodium citrate containing 0.01% Tween 20. The synthesized AuNPs were observed by TEM (JEM-2000EX II; JEOL Ltd, Tokyo, Japan). Their hydrodynamic size distribution and zeta potential was also characterized by DLS (Zetasizer Nano-ZS; Malvern Instruments Ltd, Worcestershire, UK).

PEGylation of AuNPs was conducted using PEG-SH according to a previously described method^40^. Thiol-terminated PEG was added at a concentration of 10.8 PEG/nm^2^ to the synthesized AuNP solutions. The reaction solution was maintained at 60 °C for 1 h, followed by washing three times with pure water via centrifugation. The density of conjugated PEG was determined by measuring the concentration of un-reacted PEG-SH using Ellman’s assay, according to a previously described method^40^. After the reaction, the resultant solution was centrifuged for 45 min at 45,000 rpm for 3 nm AuNPs, 10,000 rpm for 15 nm AuNPs, and 1400 rpm for 120 nm AuNPs. The supernatants were collected and the free PEG-SH concentration in them was determined using Ellman’s assay, according to the manufacturer’s instructions. Then, the amount of grafted PEG-SH was determined by subtracting the amount of free PEG-SH from the initial amount. The amount of grafted PEG was divided by the total surface area of AuNPs to obtain the surface grafting density.

### Cell viability assay

The cytotoxicity of the obtained AuNPs was evaluated via cell viability assay using human umbilical vein cell (HUVEC). The cells were grown and maintained in EGM-2 Bullet Kit medium at 37 ℃ in 5% CO_2_. The cells were seeded on a 24-well plate with an initial density of 50,000 cells/well and incubated at 37 °C in 5% CO_2_ overnight. Then, the cells were treated with 3, 15, and 120 nm AuNPs with various concentrations. After 24 h, the cells were washed with PBS, and then 500 μl medium containing 10% v/v Cell Counting Kit solution was added to each well. After 2 h of incubation at 37 ℃ in 5% CO_2_, the absorbance at 450 nm was measured using a plate reader (200 ARVO V3; PerkinElmer, Waltham, MA, U.S.A.) and normalized to that of four control wells containing only medium to assess cell viability.

### Construction of the in vitro BBB model

bEND.3 cells were seeded on the bottom side of a Transwell culture insert at a density of 3.2 × 10^4^ cells per well. The cells were grown in DMEM containing 10% inactivated FBS and 1% penicillin–streptomycin–amphotericin B and maintained in a humidified incubator at 37 °C with 5% CO_2_. Prior to the experiment, the cells were cultured for four days to become fully confluent. The culture insert was then set on a handmade culture well that was connected to a water bath, through which it could be exposed to FUS (Fig. S1). The chamber was filled with 100 μl of culture medium, the maximum volume, to avoid the reflection of ultrasound from the liquid/air interface. A further 600 μl of culture medium was added to the reservoir, which was the minimum volume required to fill the well for maximizing the concentration of permeated NPs. Clinically approved Sonazoid MBs were added to the culture well and allowed to attach to the surface of the bEND.3 cells by buoyancy. The cells were then exposed to FUS for 40 s, with burst lengths of 10 ms and a repetition frequency of 1 Hz, for 40 s using a custom-made, single element piezoelectric transducer with a focal length of 25 mm (Type C213, Fuji Ceramics, Shizuoka, Japan). The resonant frequency and acoustic pressure were 1 MHz and 88 kPa, respectively. A similar experiment was conducted but without the addition of MBs. In this case, bEND.3 cells were seeded on the upper side of the Transwell. For comparison with the previously reported BBB permeability enhancer, Reg, the BBB model was also treated with 10 μM of Reg for 30 min, instead of FUS exposure.

The time changes in TEER following the above treatments were measured using an epithelial volt-ohmmeter (EVOM2; World Precision Instruments Inc., Sarasota, FL). Immunohistochemical staining of the tight junction-associated protein ZO-1 was also conducted for bEND.3 cells before and after exposure to FUS, using anti-ZO-1 rabbit polyclonal antibodies and TRITC-conjugated anti-rabbit IgG^37^. Cell nuclei were also stained with DAPI. The cells were then observed under a confocal laser scanning microscope (TCS-SP2; Leica, Wetzlar, Germany).

### Ultrasound beam profile measurement for the in vitro setup

The ultrasound focal zone was measured at the bottom of the well filled with degassed water using a needle hydrophone (Type: 80–0.5–4.0, Imotec Messtechnik, DE). The hydrophone was stepped from − 2.4 to 2.4 mm with a 0.20-mm pitch in the X and Y directions using a three-axis motorized stage (Chuo Precision Industrial, JP) around the selected foci. An oscilloscope (HDO4024, Teledyne LeCroy, US) was connected to the hydrophone and the motorized stage to store the waveforms at each position.

### In vitro quantification of AuNPs permeation across the BBB

To evaluate the permeability of the BBB to AuNPs, immediately following treatment with FUS or Reg the culture medium in the chamber was replaced with media containing 5 × 10^5^ ppb of 3, 15, or 120 nm AuNPs. After incubation for 12 h at 37 °C with 5% CO_2_, the media in the reservoir was collected and the amount of permeated AuNPs was measured by ICP-OES (iCAP 6000; Thermo Fisher Scientific, Waltham, MA).

### FUS-induced BBB opening in a mouse brain in vivo

All animal experiments were performed in accordance with the Guidelines of Animal Experiments of the University of Tokyo, and the protocols were approved by the animal care committee of the University of Tokyo. ICR mice (8-week-old males) each weighing 35 g were purchased from CLEA Japan, Inc. (Tokyo, Japan) and housed in a 6 am−6 pm light−dark cycle. Anesthesia was induced via intraperitoneal (IP) injection of 30 mg/kg pentobarbital (Somnopenthyl) and then the head was shaved. This was followed by the intravenous (IV) injection of 100 μl AuNPs (4 mg/ml) or 400 μl trypan blue (8 mg/ml), with subsequent IV injection of 100 μl Sonazoid (8.0 × 10^8^ to 8.0 × 10^9^ bubbles/ml). Immediately after the injections, ultrasound jelly was applied to the mouse’s shaved head, which was then exposed to 1 MHz FUS for 40 s, with a burst length of 10 ms and a repetition frequency of 1 Hz for 40 s. After 4 h, the mice were euthanized by IP administration of pentobarbital (240 mg/kg). After saline perfusion to remove blood content, brain tissue was collected for the following evaluations.

### Ultrasound beam profile measurement for the in vivo setup

Ultrasound beam profiles were measured in the free-field degassed water-filled water bath using a needle hydrophone (Type: 80–0.5–4.0, Imotec Messtechnik, DE). The hydrophone was stepped from − 3.0 to 3.0 mm in the X direction and 0 mm to 12.0 mm in the Z direction with a 0.20-mm pitch by a three-axis motorized stage (Chuo Precision Industrial, JP) around the tip of the waveguide (Fig. S7). An oscilloscope (HDO4024, Teledyne LeCroy, US) was connected to the hydrophone and the motorized stage to store the waveforms at each position. The X–Y plane beam profiles were also obtained at the Z positions of 3 mm and 9 mm.

### In vivo evaluation of the delivery of AuNPs into the brain in vivo

The collected brains were fixed in 10% formalin to perform the microscopic observations. To evaluate the permeation of trypan blue, fixed tissue was sectioned using a scalpel and then observed under a stereoscopic microscope (SMZ1000, Nikon, Tokyo, Japan). The fixed tissue was also cryostat-sectioned, followed by HE and silver staining using standard techniques. The stained sections were observed under an optical microscope (BZ-9000; Keyence, Osaka, Japan). To quantify the amount of AuNPs delivered, whole brain tissue was digested in nitric acid at 80 °C for 2 days. The amount of AuNPs in the digested solution was measured using ICP-OES.

### Statistical analysis

All statistical analyses were performed via a one-way analysis of variance followed by the Tukey HSD post hoc test, using Kaleida Graph 4.0J (Hulinks, Tokyo, Japan). A value of p < 0.05 was considered to be statistically significant.

### Kinetic modeling calculation

The permeation of AuNPs through gaps opened in the BBB was calculated by integrating Eqs. 1 and 2 using the finite-difference method with a time step of 1.0 × 10^−2^ s. The accumulation of AuNPs in the brain was calculated by integrating Eqs. 10 and 11 using the finite-difference method with a time step of 1.0 × 10^−2^ s. A detailed explanation of the parameters and the model can be found in the Supporting Information.

## Supplementary information

Supplementary Information.

## Data Availability

The datasets generated analyzed during the current study are available from the corresponding author on reasonable request.
