# Supplementary material for: Investigating the optimum size of nanoparticles for their delivery into the brain assisted by focused ultrasound-induced blood–brain barrier opening
Source: Sci Rep. 2020 Oct 26;10:18220. doi: 10.1038/s41598-020-75253-9 (PMC7588485; doi:10.1038/s41598-020-75253-9)
Supplement: Supplementary file 1 — Supplementary Information. [file 41598_2020_75253_MOESM1_ESM.pdf]

Supplementary Information

Investigating the optimum size of nanoparticles for their delivery into the brain assisted by focused ultrasound-induced blood–brain barrier opening

Seiichi Ohta,[1*] Emi Kikuchi,[2] Ayumu Ishijima,[3] Takashi Azuma,[1,2] Ichiro Sakuma,[2,3] Taichi Ito[1,2]

1 Center for Disease Biology and Integrative Medicine, The University of Tokyo, 7-3-1 Hongo, Bunkyo-ku, Tokyo 113-8655, Japan
2 Department of Bioengineering, The University of Tokyo, 7-3-1 Hongo, Bunkyo-ku, Tokyo 113-8656, Japan
3 Department of Precision Engineering, The University of Tokyo, 7-3-1 Hongo, Bunkyo-ku, Tokyo 113-8656, Japan

* Corresponding author
E-mail: s-ohta@sogo.t.u-tokyo.ac.jp
Current address: Institute of Engineering Innovation, The University of Tokyo, 7-3-1 Hongo, Bunkyo-ku, Tokyo 113-8656, Japan

Table of Contents

Supporting Methodology

Modeling of closing kinetics of opened BBB gaps 3

Modeling of size-dependent blood half-lives of nanoparticles 3

Parameters used for the model calculation 4

Supporting Figures and Tables

Figure S1. Apparatus for the FUS-induced enhancement of BBB permeability to nanoparticles in vitro 5

Figure S2. Ultrasound beam profile at the bottom of the well for the *in vitro* FUS-exposure setup. 5

Figure S3. Time change of TEER induced by FUS exposure without MBs 6

Figure S4. Hydrodynamic size distribution of the synthesized AuNPs as measured by DLS........ 6

Figure S5. Cell viability of HUVECs incubated with 3, 15, and 120 nm AuNPs for 24h........ 7

Figure S6. The quantity of 3, 15, and 120 nm AuNPs that permeated through the *in vitro* BBB model following FUS exposure without MBs........ 7

Figure S7. Apparatus for in vivo BBB opening via intracranial FUS exposure........ 8

Figure S8. An ultrasound beam profile for the *in vivo* FUS exposure setup. (a) Apparatus used for the ultrasound beam profile measurement. (b) An ultrasound beam profile obtained by the measurement. Definitions of the X, Y, and Z axes are shown in (a)........ 9

Figure S9. Brain sections of mice subjected to FUS with higher MB doses (3.0×10^8 bubbles/mouse)...... 10

Figure S10. Severe bleeding following FUS exposure with acoustic pressure and/or an MB dose that was too high 10

Figure S11. The effect of acoustic pressure on the amount of 3 nm AuNPs delivered into the brain assisted by FUS-induced BBB opening........ 11

Figure S12. The amount of 3, 15, and 120 nm AuNPs delivered into the brain assisted by FUS-induced BBB opening with an MB dose (3.0×108 bubbles/mouse)........ 11

Figure S13. Effect of k_{rg} and A_{k0} on the size-dependent BBB permeation of AuNPs 12

Figure S14. Previously reported data and theoretical fitting of size-dependent blood half-lives of nanoparticles in mice. 12

Figure S15. Effect of (a) k_{rg} and (b) A_{k0} on the size-dependent delivery of nanoparticles into the brain when assisted by FUS 13

Table S1 Summary of the characterization of synthesized AuNPs 14

Table S2. Summary of parameters used for the model calculation 14

Supporting Methodologies

Modeling of closing kinetics of opened BBB gaps

Experimentally, it was difficult to observe changes over time in the size of the gaps that opened in the BBB, r_g, so we assumed a simple first-order kinetic law to describe the closing kinetics of the gaps as follows:

$$r_g = r_{g0} \exp\left(-k_{rg} t\right) \qquad \text{(eq. S1)}$$

, where r_{g0} and k_{rg} represent the size of the BBB gaps that opened immediately after FUS exposure and the rate constant for the gap closing, respectively.

Accompanying the decrease in r_g, the pore density, A_k also decreases over time. Assuming that the number of pores, N, does not change during the recovery and that the size of each gap decreases equally following eq. S1, the changes in A_k can be described as follows:

$$A_k = \frac{N\pi r_g{}^2}{A_m} = \frac{N\pi\left(r_{g0} \exp\left(-k_{rg} t\right)\right)^2}{A_m} = \frac{N\pi r_{g0}{}^2}{A_m} \exp\left(-2k_{rg} t\right)$$

$$= A_{k0} \exp\left(-2k_{rg} t\right) \qquad \text{(eq. S2)}$$

, where A_m and A_{k0} are the total area of the cellular sheet and the pore density immediately after FUS exposure, respectively. Since TEER recovered to the initial value in ca. 4 h (Figure 2b), k_{rg} was determined to be 5.6×10^{-5} s^{-1}, so that A_k becomes 20% of A_{k0} after 4 h.

Modeling of size-dependent blood half-lives of nanoparticles

We compiled previously reported blood half-lives of PEGylated nanoparticles, regardless of the type of materials used, [1-5] and then plotted against their size (Figure S13). It was found that the size-dependency of the blood half-lives of particles can be fitted with the following two master curves:

$$\tau_1 = a_1 \exp\left(b_1 t\right) \qquad \text{(eq. S3)}$$

$$\tau_2 = a_2 \exp\left(-b_2 t\right) \qquad \text{(eq. S4)}$$

, where τ_1 corresponds to blood half-lives which are dominated by renal clearance, while τ_2 corresponds to blood half-lives which are dominated by RES uptake. a_1, a_2, b_1, and b_2 are the

fitting parameters. a_1 = 2.0×10^{-3} [s], a_2 = 4.0×10 [s], b_1 = 1.4 [s^{-1}], and b_2 = 2.2×10^{-2} [s^{-1}] were obtained by fitting eqs. S3 and S4 to the experimental data using the least square method (Figure S13). For each particle size, τ_1 and τ_2 were calculated and then the smaller of the values was used as the blood half-life, τ (r_{NP}). By using τ (r_{NP}) obtained in this way, a size-dependent elimination-rate constant $k_{el}(r_{NP})$ could be calculated as follows:

$$k\left(r_{NP}\right)=\frac{\ln 2}{\tau\left(r_{NP}\right)} \qquad \text{(eq. S5)}$$

Parameters used for the model calculation

Parameters used for the model calculation are summarized in Table S2.

Supporting Figures and Tables

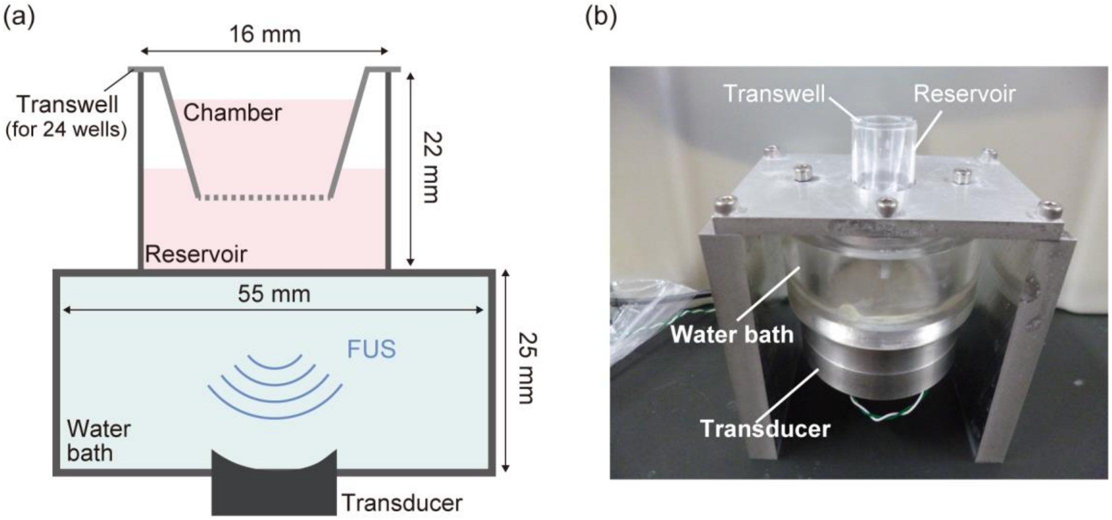

Figure S1. (a) A schematic diagram and (b) a photograph of the apparatus used for the evaluation of FUS-induced enhancement of BBB permeability to nanoparticles *in vitro*.

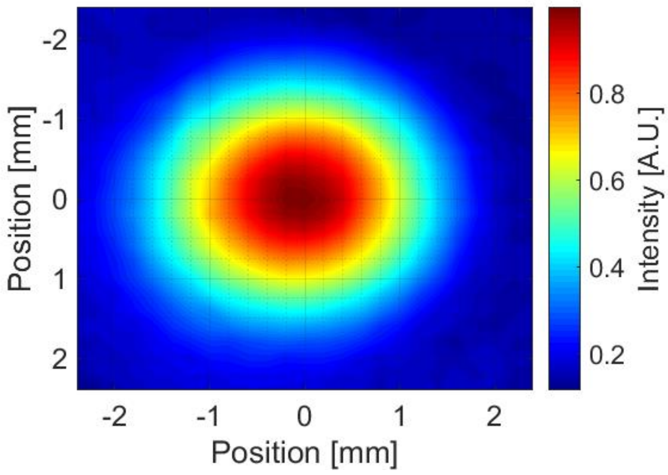

Figure S2. Ultrasound beam profile at the bottom of the well for the *in vitro* FUS-exposure setup.

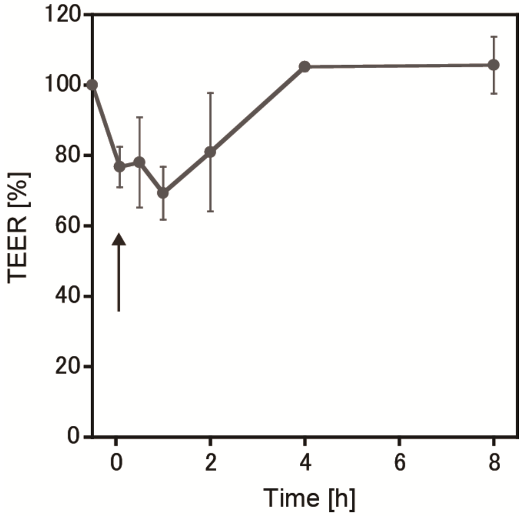

Figure S3. Time change of TEER induced by FUS exposure without MBs. An acoustic pressure of 320 kPa was used. The arrow indicates the time when FUS exposure was performed (defined as 0 h). N = 4

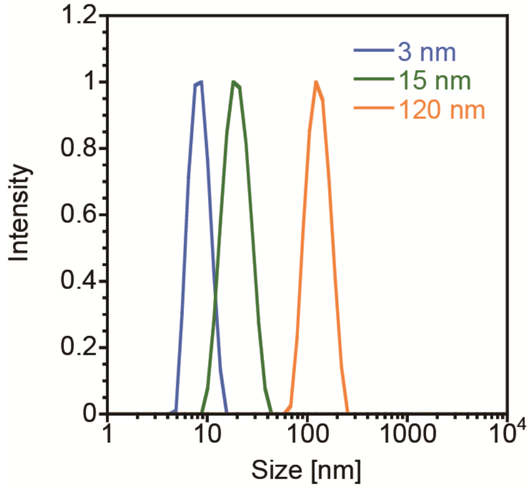

Figure S4. Hydrodynamic size distribution of the synthesized AuNPs as measured by DLS.

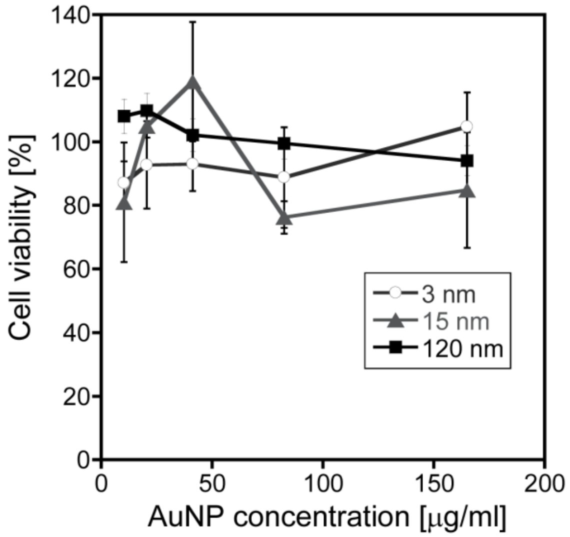

Figure S5. Cell viability of HUVECs incubated with 3, 15, and 120 nm AuNPs for 24h.

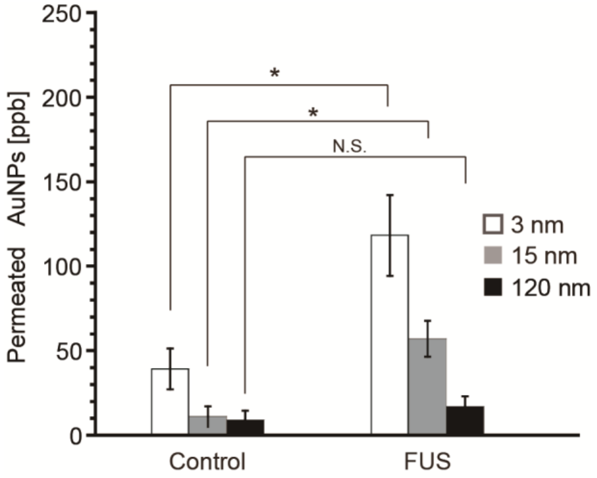

Figure S6. The quantity of 3, 15, and 120 nm AuNPs that permeated through the *in vitro* BBB model following FUS exposure without MBs. An acoustic pressure of 320 kPa was used. * $p < 0.05$; N.S. not significant. N = 4.

(a) (b)

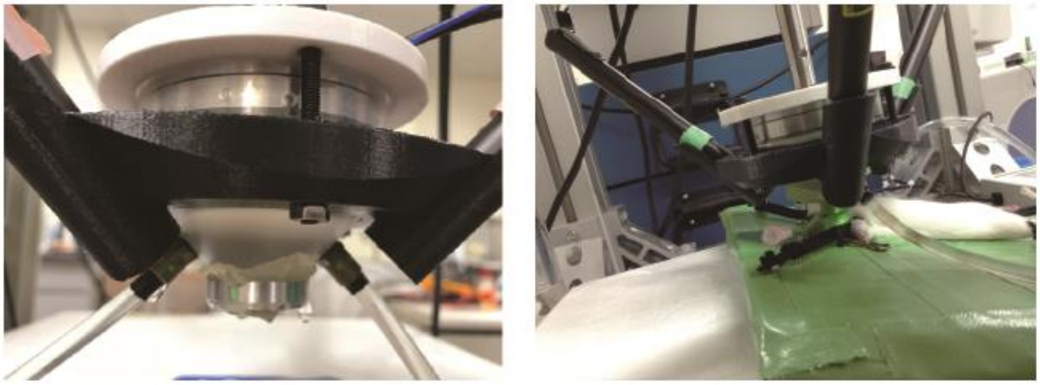

Figure S7. Apparatus for the *in vivo* investigation of BBB opening following intracranial FUS exposure. (a) The transducer used for intracranial FUS exposure. (b) A representative image showing intracranial FUS exposure in mice.

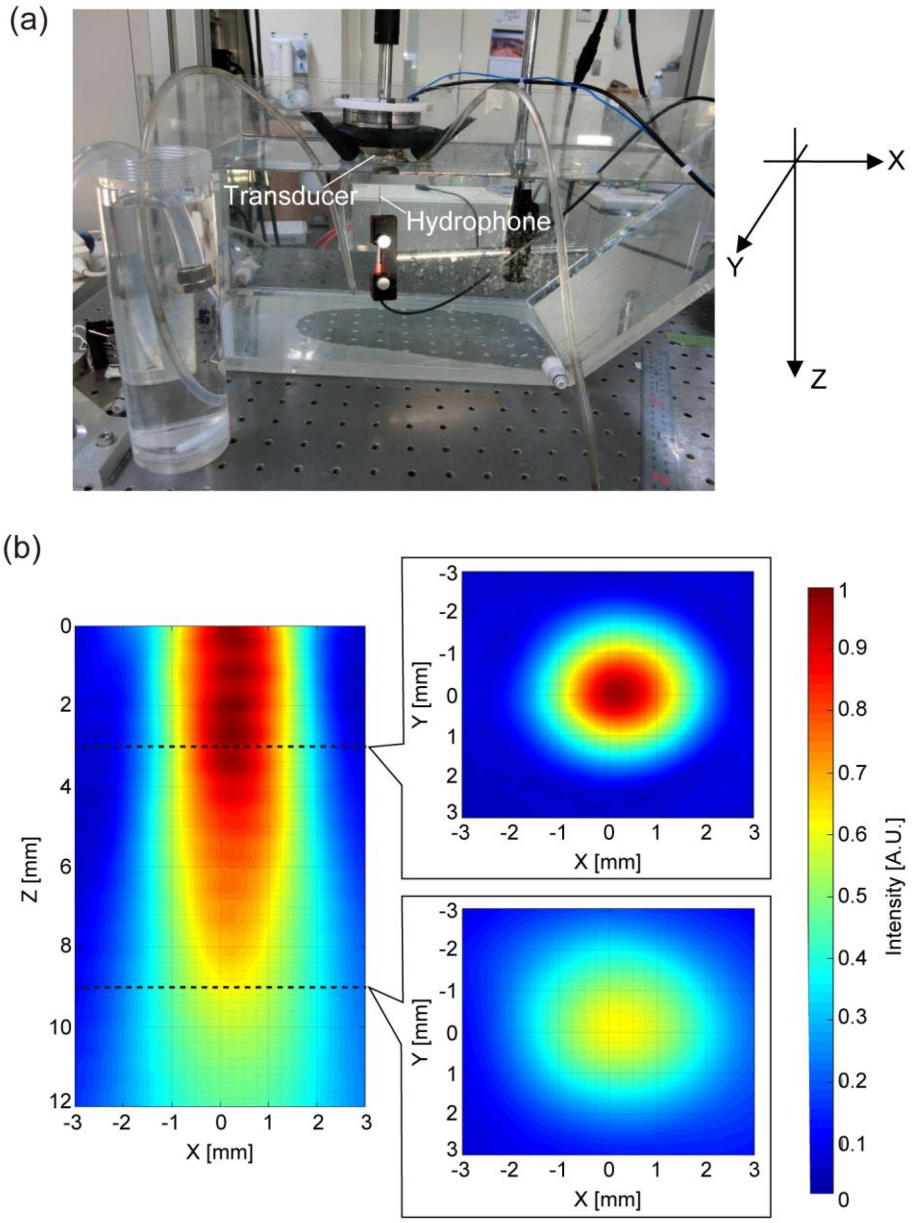

Figure S8. An ultrasound beam profile for the *in vivo* FUS exposure setup. (a) Apparatus used for the ultrasound beam profile measurement. (b) An ultrasound beam profile obtained by the measurement. Definitions of the X, Y, and Z axes are shown in (a).

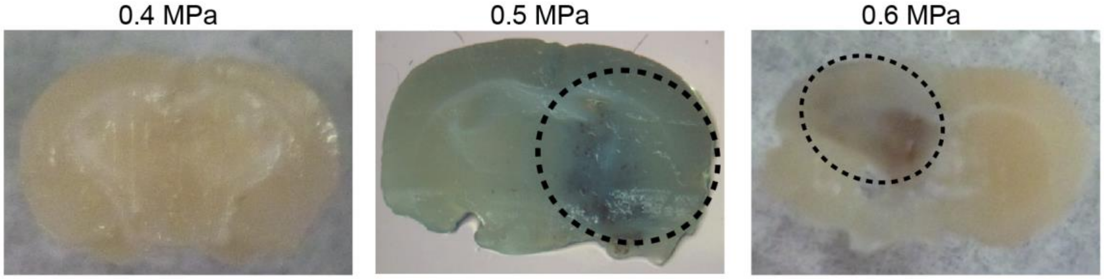

Figure S9. Brain sections of mice subjected to FUS with higher MB doses (3.0×10^{8} bubbles/mouse). Dotted circles indicate areas where the permeation of trypan blue was observed.

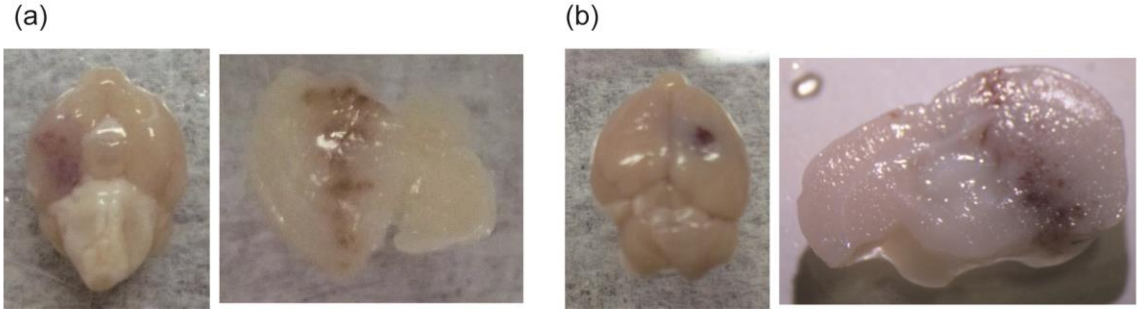

Figure S10. Severe bleeding following FUS exposure with acoustic pressure above 0.8 MPa. MB doses was (a) 8.0×10^{7} bubbles/mouse (b) 3.0×10^{8} bubbles/mouse.

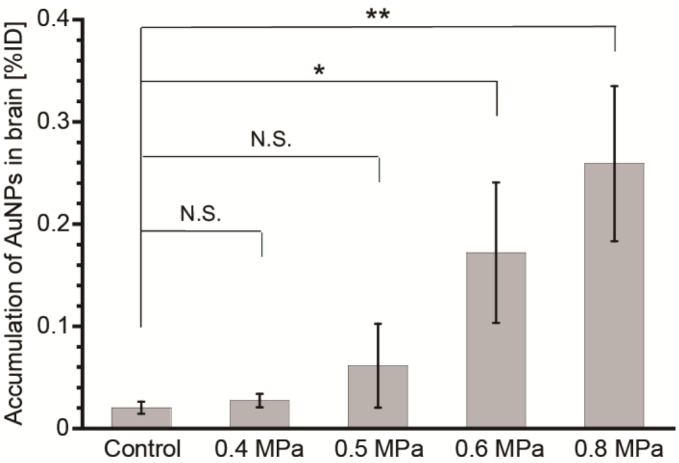

Figure S11. The effect of acoustic pressure on the amount of 3 nm AuNPs delivered into the brain assisted by FUS-induced BBB opening. The dose of MBs was 3.0×10^{8} bubbles/mouse. ** $p < 0.01$; * $p < 0.05$; N.S. not significant. N = 4.

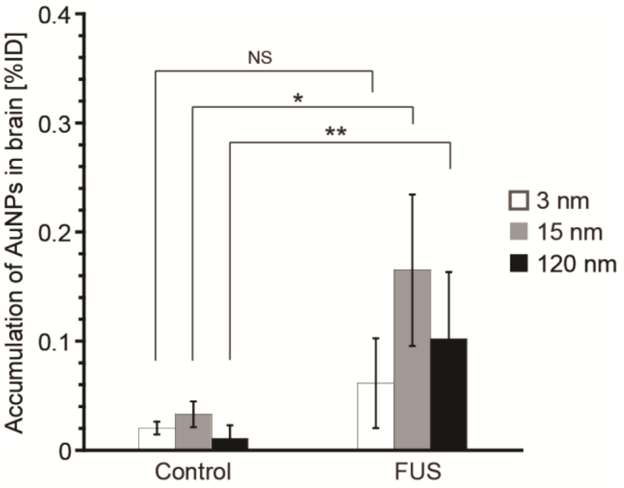

Figure S12. The amount of 3, 15, and 120 nm AuNPs delivered into the brain assisted by FUS-induced BBB opening with an MB dose of 3.0×10^{8} bubbles/mouse. The acoustic pressure was 0.5 MPa. ** $p < 0.01$; * $p < 0.05$; N.S. not significant. N = 4.

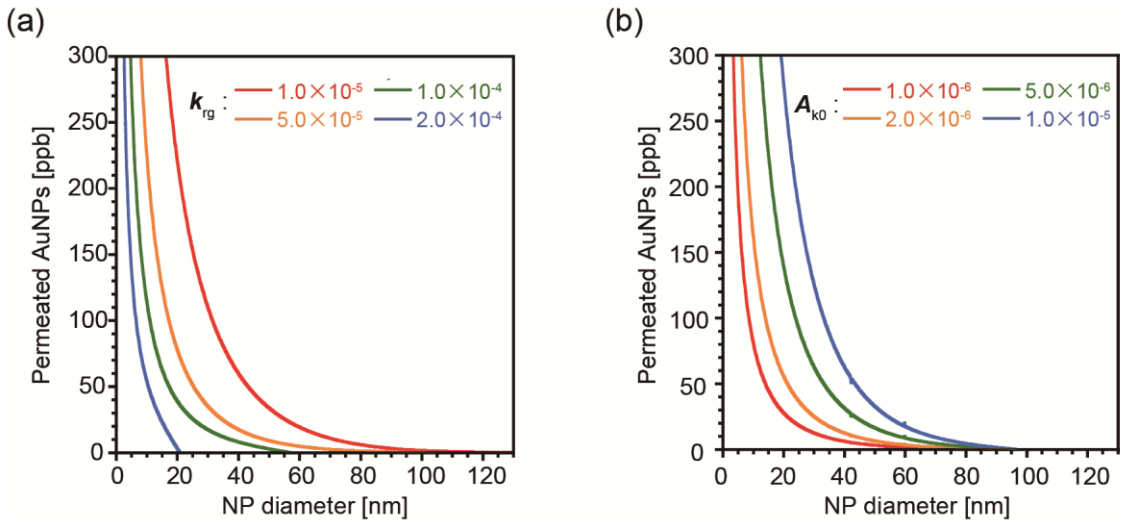

Figure S13. Effect of (a) k_{rg} and (b) A_{k0} on the size-dependent BBB permeation of AuNPs, as calculated based on a modified pore-flow model. The size of the gaps which opened in the BBB was assumed to be 100 nm.

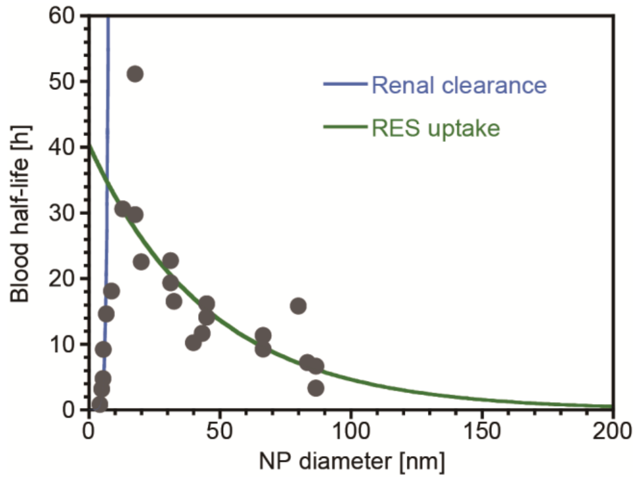

Figure S14. Effect of the size of nanoparticles on their blood half-lives in mice. Plots represent the previously reported blood half-lives of PEGylated nanoparticles, regardless of the type of material used.[1-5] Solid lines represent the fitted lines of the experimental plots obtained from eqs. S3 (blue) and S4 (green).

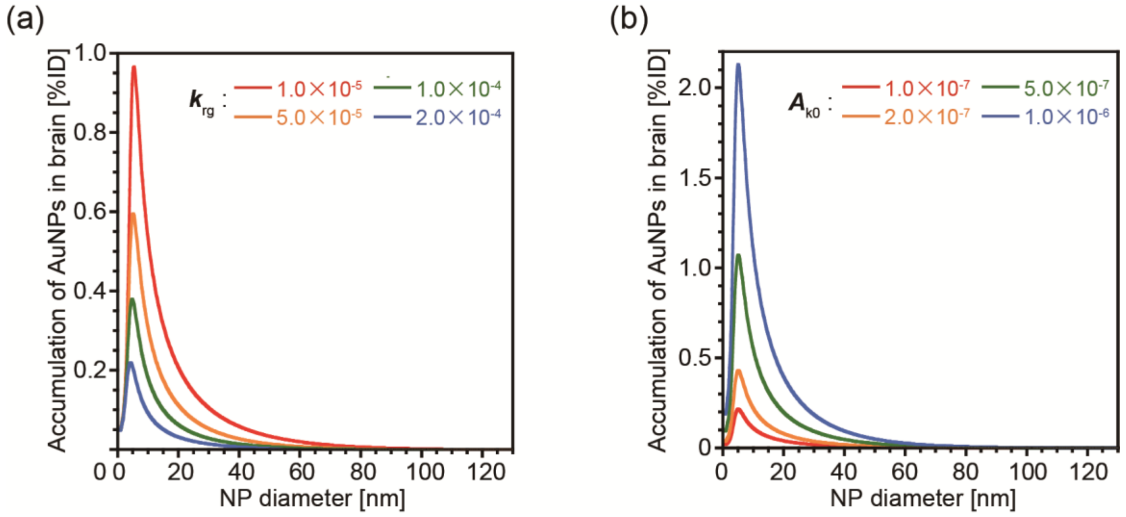

Figure S15. Effect of (a) k_{rg} and (b) A_{k0} on the size-dependent delivery of nanoparticles into the brain when assisted by FUS and calculated by coupling the BBB permeability model with nanoparticle elimination kinetics. The size of the gaps which opened in the BBB was assumed to be 100 nm.

Table S1 Summary of the characterization of synthesized AuNPs

Size [nm][a]	Zeta potential [mV]		Surface density of conjugated PEG [/nm^2][b]
	Before PEGylation	After PEGylation	
3.7 ± 0.5	−34	−9	1.1
14.4 ± 1.7	−28	−7	1.6
120 ± 7.2	−38	−6	1.3

[a] Diameter determined from TEM images

[b] Surface density of conjugated PEG estimated via Ellman's assay.

Table S2. Summary of parameters used for the model calculation

Parameter		Value	Note
Volume of chamber	V_{in}	100 μl	
Volume of reservoir	V_{out}	600 μl	
Area of semipermeable membrane	A_m	3.3×10^{-5} m^2	
Volume of the brain	V_b*	7.0×10^{-7} m^3	From ref. 6
Volume of whole blood	V_{pl}*	2.8 ml	From ref. 7
Area of blood vessels in the brain	A_b*	1.2×10^{-2} m^2	From ref. 8
Boltzmann constant	K_b	1.38×10^{-23} J/K	
Temperature	T	298 K	
Viscosity	η	8.9×10^{-4} Pa s	
Thickness of the BBB	Δx	100 nm	From ref. 9
Rate constant for gap closing	k_{rg}	5.6×10^{-5} s^{-1}	Estimated based on TEER
Rate-constant parameter for elimination	a_1	2.0×10^{-3} s	Estimated by fitting to Fig. S11
Rate-constant parameter for elimination	a_2	4.0×10 s	Estimated by fitting to Fig. S11
Rate-constant parameter for elimination	b_1	1.4 s^{-1}	Estimated by fitting to Fig. S11
Rate-constant parameter for elimination	b_2	2.2×10^{-2} s^{-1}	Estimated by fitting to Fig. S11
Initial density of gaps opened in the BBB	A_{k0}	2.5×10^{-6} (*in vitro*)<br>2.6×10^{-7} (*in vivo*)	Fitting parameter

* V_b, V_{pl}, and A_b in mice were estimated via a body-weight based extrapolation from humans, assuming that the body weights of humans and mice are 60 kg and 35 g, respectively.

References

1. Choi, H. S.; Liu, W.; Misra, P.; Tanaka, E.; Zimmer, J. P.; Itty Ipe, B.; Bawendi, M. G.; Frangioni, J. V. Renal clearance of quantum dots. *Nat. Biotechnol.* **2007,** *25*, 1165-70.
2. Perrault, S. D.; Walkey, C.; Jennings, T.; Fischer, H. C.; Chan, W. C. W. Mediating Tumor Targeting Efficiency of Nanoparticles Through Design. *Nano Lett.* **2009,** *9*, 1909-1915.
3. Cho, W. S.; Cho, M.; Jeong, J.; Choi, M.; Cho, H. Y.; Han, B. S.; Kim, S. H.; Kim, H. O.; Lim, Y. T.; Chung, B. H.; Jeong, J. Acute toxicity and pharmacokinetics of 13 nm-sized PEG-coated gold nanoparticles. *Toxicol. Appl. Pharmacol.* **2009,** *236*, 16-24.
4. Hong, G.; Robinson, J. T.; Zhang, Y.; Diao, S.; Antaris, A. L.; Wang, Q.; Dai, H. In vivo fluorescence imaging with Ag2S quantum dots in the second near-infrared region. *Angew. Chem. Int. Ed. Engl.* **2012,** *51*, 9818-21.
5. Zhang, G.; Yang, Z.; Lu, W.; Zhang, R.; Huang, Q.; Tian, M.; Li, L.; Liang, D.; Li, C. Influence of anchoring ligands and particle size on the colloidal stability and in vivo biodistribution of polyethylene glycol-coated gold nanoparticles in tumor-xenografted mice. *Biomaterials* **2009,** *30*, 1928-36.
6. Allen, J; Damasio, H.; Grabowski T. J.; Normal Neuroanatomical Variation in the Human Brain: An MRI-Volumetric Study. *Am. J. Phys. Anthropol.* **2002**, 118, 341-58.
7. Riches A. C.; Sharp J. G.; Thomas D. B.; Smith V. Blood volume determination in the mouse. *J. Phisiol.* **1973**, 228, 279-84.
8. Pardridge W. M. Blood–brain barrier delivery. *Drug Discov. Today* **2007**, 12, 54-61.
9. Wong, A. D.; Ye M.; Levy A. F.; Rothstein J. D.; Bergles D. E.; Searson P. C. The blood-brain barrier: an engineering perspective. *Front. Neuroeng*. **2013**, 6, 1-22.
